# Temporal and spatiotemporal investigation of tourist attraction visit sentiment on Twitter

**DOI:** 10.1371/journal.pone.0198857

**Published:** 2018-06-14

**Authors:** Jose J. Padilla, Hamdi Kavak, Christopher J. Lynch, Ross J. Gore, Saikou Y. Diallo

**Affiliations:** 1 Virginia Modeling Analysis and Simulation Center, Old Dominion University, Suffolk, Virginia, United States of America; 2 Modeling Simulation and Visualization Engineering Department, Old Dominion University, Suffolk, Virginia, United States of America; Tampere University of Technology, FINLAND

## Abstract

In this paper, we propose a sentiment-based approach to investigate the temporal and spatiotemporal effects on tourists’ emotions when visiting a city’s tourist destinations. Our approach consists of four steps: data collection and preprocessing from social media; visitor origin identification; visit sentiment identification; and temporal and spatiotemporal analysis. The temporal and spatiotemporal dimensions include day of the year, season of the year, day of the week, location sentiment progression, enjoyment measure, and multi-location sentiment progression. We apply this approach to the city of Chicago using over eight million tweets. Results show that seasonal weather, as well as special days and activities like concerts, impact tourists’ emotions. In addition, our analysis suggests that tourists experience greater levels of enjoyment in places such as observatories rather than zoos. Finally, we find that local and international visitors tend to convey negative sentiment when visiting more than one attraction in a day whereas the opposite holds for out of state visitors.

## Introduction

Tourism is one of the most important source of economic activities in cities and a significant item in the global economy [1]. For many cities and countries tourism is crucial for economic sustainability and development [2]. For instance, US cities like Las Vegas and Orlando have become some of the fastest developing metropolitan areas and many other cities are following suit [3]. As of January 2018, the US Bureau of Labor and Statistics states that [4] Las Vegas area employment numbers are 29% leisure- and hospitality-related followed by 17% Trade/Transportation/Utilities and 14% Professional/Business Services, which are indirectly impacted by tourism.

As tourism becomes crucial to the growth of cities and countries, understanding tourists’ behaviors provides further insight into how to increase tourists’ satisfaction with their visits and gain loyal return visitors as a result [5]. A considerable amount of research has been devoted to understanding and measuring visitors’ satisfaction and providing insights for policymaking [6–9]. In this respect, is important for the growth of these economies to understand tourism trends, the locations that tourists visit, and the opinions that visitors form based on their choices.

Traditionally, researchers rely on surveys [10] and time-space travel diaries [11] to gain an understanding of the trends, visitors’ location choices, and visitors’ opinions. Sometimes, these data collection techniques are complemented with digital devices that track locations over time [12, 13]. While common, these techniques are costly, require active participation, and need to be repeated periodically to understand and measure changes over time.

The advent of social media and the prevalence of mobile devices have created new ways to know the locations that people visit and what they like. Social media services such as Foursquare and Twitter allow users to report their locations with high precision. When these participatory data are made public, they provide unique insights that are challenging to identify with traditional survey-based analysis techniques due to data size, recentness, and geographic scale. Public data collected from Twitter [14] and Flickr [15] data can be used for understanding peoples’ movement patterns; illustrating that social media data provides a longitudinal dimension that is lacking within transactional surveys. Studies like [16] and [17] in exploring spatiotemporal tourist patterns, [18] in extracting tourist attraction visits and visit lengths, [19] in estimating visitation numbers, [20] in identifying behaviors and attributes, and [21, 22] in identifying home locations, highlight the range of contributions that social media data provides for understanding tourists’ behaviors.

One element often overlooked in the literature is capturing the ‘emotions’ of tourists especially across dimensions like visitors’ origins, locations, and time. Emotions about places and day/time have implications on tourists’ destination loyalty and effects their sharing of positive or negative information with others. Recurring visits and positive/negative information about a place or city’s tourist amenities end up affecting tourism-oriented companies’ revenues, employment, and investment decisions in the future, among others. Sentiment analysis is a technique used to extract emotions and feelings from unstructured text [23]. It has been successfully applied to a variety of domains [24–27]. However, within the tourism domain, studies mostly focus on broad topics such as online hotel reviews [28, 29] or on tourists’ perceptions about cities [30, 31]. A more comprehensive, multi-dimensional view of tourists’ perspectives can better inform decision makers or fellow tourists.

In this paper, we propose a method to investigate tourists’ behaviors related to attraction visits on temporal (day, season, and weekday/weekend) and spatiotemporal (sentiment progression, enjoyment, and multi-location sentiment progression) characterizations. Visitors’ origins (local, out of state, and international) provide further information along the temporal and spatiotemporal dimensions. Details of our method, the use-case, and results and discussion are presented in the remainder of the paper.

## Method

**Fig 1:**
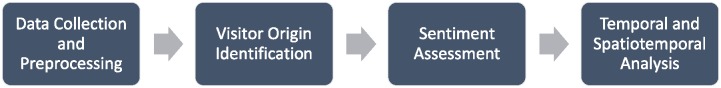
Method summary. The summary of our 4-step method containing the steps of data collection and preprocessing, visitor origin identification, visit sentiment identification, and temporality inspection.

The proposed approach consists of four major steps summarized in Fig 1. The first step is data collection and preprocessing in which attraction and visit datasets for the selected city are constructed and cleaned. The second step is the identification of visitors’ origins based on their self-reported locations. The third step is sentiment assessment that identifies positive and negative emotions in all tweets pertaining to attraction visits. The last step investigates attraction visit tweets according to several temporal and spatiotemporal dimensions and reports on the identified insights.

### Data collection and preprocessing

Data collection consists of first compiling a list of tourist attraction names and related information for a selected city and then collecting geo-located Twitter messages corresponding to that city. The tourist attraction list is obtained by searching popular attractions using online tourist platforms. We use TripAdvisor [32] to obtain the overall rankings of places to understand their popularity and we use Timeout [33] and Yelp [34] to obtain complementary information. The attraction list contains a comprehensive list of attributes including *name*, *category (e.g., museum)*, *opening/closing times*, *keywords*, and *geographical boundary*. To simplify the boundary identification process, we implemented a web-based tool that stores clicked points as latitude and longitude pairs [35]. Keywords are a collection of words to indicate an attraction using its current names, old names, alternative names, category, and potential misspellings of the names and the category.

We collect geo-located Twitter messages using the Streaming API [36] by specifying the selected city’s geographical boundary limits. This data consists of all tweets shared within the city regardless of their content. To improve the quality of the resulting dataset, we eliminate tweets shared by non-human accounts. We first rely on the number of messages and links within the Twitter messages because non-human accounts tend to have high numbers for both [37]. Then, we create a user profile containing the average number of tweets per day and the ratio of links over total tweets. We further provide the ratio of links shared from popular social media services (e.g., Foursquare) because we aim to capture and keep the users that automatically post their messages on Twitter through other social media services. Lastly, we eliminate users with unrealistically high average number of tweets per day (over 100) and high link percentage (90%) through the Twitter platform but not through other social media services. This process eliminated approximately 13% of the identified users.

Data preprocessing involves applying a series of algorithms on the collected data to identify tweets that indicate a visit to an attraction. To identify whether a person visited an attraction, we first look for tweets shared within the boundary of the attraction and find those with tweet text that mentions at least one keyword of the attraction. By collectively considering a tweet’s text and attraction boundaries, we identify tweets that are more likely to be related to attraction visits. In fact, a cloud visualization of tweets after keyword-based filtering reveals a picture that is populated with attraction visit-related words demonstrating its merit. Previous studies such as [38] consider all tweets within an attraction boundary as attraction visits. However, such an elementary approach would capture tweets which may not relate to attraction visits. We then collect all of the tweets shared over the following six hours of an attraction visit for each of the identified visitors. At this point, we do not further examine tweet content as we have already identified that the person is visiting the attraction. Lastly, we identify tweets originating within a one-kilometer radius of an attraction which mention the attraction’s full name. This step identified tweets that are not captured by boundary-based approaches. We then eliminate tweets that are shared outside of each attractions’ regular business hours. We note that our data collection and use practices comply with Twitter’s terms of service.

### Visitor origin identification

We identify the origins of users that visit at least one attraction. Visitor origin is an important factor because there is a likelihood that, for instance, international visitors have different attraction visitation patterns and experience different emotions than local visitors who have seen their local attractions many times. Visitor origin refers to the current and long-term residing locations of visitors. We use this to establish whether differences exist within visitation and sentiment patterns with respect to visitor origin. We introduce three types of visitor origins that captures different profiles of attraction visitors.

- *Local:* visitors residing in the state or metropolitan area containing the selected city.
- *Out of state:* domestic visitors residing anywhere in the country containing the selected city that do not qualify as local visitors.
- *Internationals:* visitors coming from another country.

### Sentiment assessment

Sentiment plays the role of opinion or emotion about a location, time, or visitor type. We use the SentiStrength sentiment analysis algorithm [39] that brings an algorithmic approach to sentiment analysis with considerations for short and informal texts and has been successfully applied in topics ranging from engagement in communities [40] to investigation of information dissemination [41]. [42] shows that SentiStrength performs as good as the state-of-the-art sentiment analysis algorithms for Twitter data. The SentiStrength algorithm is summarizable in two steps [43]:

*Step 1*: Sentiment term scoring is repeated only once during training of the machine learning classifier.

- The algorithm starts with a manually identified positive and negative stemmed terms list with each term’s strength score identified by humans.
- The initial term list is then used in training a machine learning classifier to modify each term’s strength score and to check whether the new score improves the overall accuracy of the classifier. This process repeats until accuracy no longer increases.

*Step 2*: Sentiment calculation repeats whenever new sentiment needs to be calculated for sentences that were not used for training the classifier.

- Text is separated into sentences which are then tokenized into stemmed words.
- Each word is assigned a score based on the initial term list used in the classifier.
- Additional rules are applied to deal with issues present within informal text including, spelling corrections, idioms, and the translation of emoticons.
- All calculated scores are summed for negatives and positives. The result is presented in the form of categories (e.g., negative) or scales (e.g., -3).

As a result of these two steps, each tweet in our dataset is assigned an integer-based sentiment score between -4 and +4 representing extremely negative to extremely positive opinions.

### Temporal and spatiotemporal analysis

We inspect three temporality factors and conduct analysis. We first inspect **the day of the year** to provide a picture of the daily attraction visit sentiment for the city. To capture both positive and negative sentiment changes throughout the year, we propose the use of daily sentiment percentages. We generate a time series data set $D=\{d_{1},d_{2},\cdots ,d_{n}|d_{x}=\left\{d_{x}^{+},d_{x}^{o},d_{x}^{-}\right\}\}$ where $d_{x}^{+}=\frac{\#\;of\;positive\;tweets\;on\;day\;x}{\#\;of\;total\;tweets\;on\;day\;x}$, $d_{x}^{o}=\frac{\#\;of\;neutral\;tweets\;on\;day\;x}{\#\;of\;total\;tweets\;on\;day\;x}$, and $d_{x}^{-}=\frac{\#\;of\;negative\;tweets\;on\;day\;x}{\#\;of\;total\;tweets\;on\;day\;x}$. We inspect D visually and identify and explain outstanding patterns. Second, we examine the meteorological **season** [44] by calculating the daily score based on $S_{x}=\frac{\sum tweet\;sentiment\;scores\;on\;day\;x}{\#\;of\;total\;tweets\;on\;day\;x}$, grouping the scores according to seasons, and inspect the resulting distribution. Third, we look at the **day of the week**. This is very similar to the season inspection except that the grouping occurs at the day of the week level.

**Fig 2:**
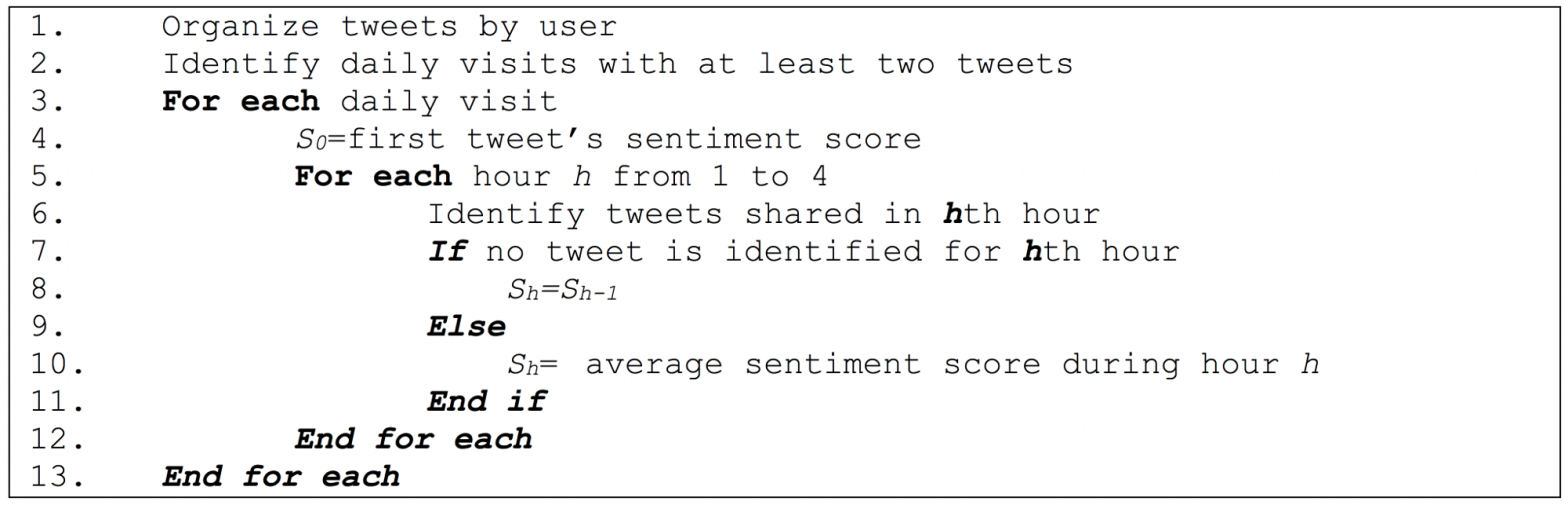
Visit length sentiment algorithm. Pseudo code for capturing the progress of sentiment over time.

For the spatiotemporal analysis, we look at the **location sentiment progression** and its effect on attraction visit sentiment. The algorithm shown in Fig 2 generates sentiment changes according to visit length. The algorithm identifies attraction visits with at least two tweets shared during the same day and then traces the average sentiment progression for that visitor’s tweets over the following four hours. Then, the results are aggregated by the initial sentiment scores (positive, neutral, negative). As a result, we separately capture how people feel based on their initial feelings.

Regardless of the sentiment progression trend, it is challenging to compare sentiment progression measurements because of (1) the varying magnitude of trends, (2) the existence of three different lines (positive, negative, and neutral), and (3) the different weights carried by different visit hours (i.e., different ratio of tweet shares). To simplify the comparison, we propose an **enjoyment measure** to address these challenges. We start by simplifying the magnitude of tweets by calculating the mean of positive (*s*_*pos*_) and negative scores (*s*_*neg*_) by hour $\bar{s}=\frac{s_{pos}+s_{neg}}{2}$. We then normalize this with the size of the difference between positive and negative scores $\tilde{\bar{s}}=\frac{\bar{s}}{s_{pos}-s_{neg}}$. This provides scores per hour on the graph. Next, we provide a weighting function (*f*_*w*_) to capture the ratio of sentiment expressions over time through a piecewise exponential function shown in Eq (1). We sum all individually weighted scores to calculate our enjoyment measure in Eq (2).

$$\begin{array}{c} f_{w}\left(x\right)=\left\{ \begin{array}{cc} 0.352537e^{0.2407x} & x<2\\ 0.409835e^{-0.6544x} & x\geq 2 \end{array}\right. \end{array}$$ (1)

$$\begin{array}{c} f_{em}=\sum_{h=0}^{4}\frac{s_{pos}+s_{neg}}{2(s_{pos}-s_{neg})}f_{w}\left(h\right) \end{array}$$ (2)

The last spatiotemporal factor we inspect is the relationship between visiting multiple attractions and their associated sentiment scores (**multi-location sentiment progression**). This factor provides insight on how visiting multiple places affect overall trip enjoyment by examining sentiment trends from people visiting multiple attractions in a day. We first investigate how the average sentiment scores per visit are distributed and inspect trends in subsequent visits. We do this by conducting linear regression analysis with respect to scores in increasing number of visits.

Table 1 summarizes the sentiment-based temporal and spatiotemporal parameters to be assessed through the proposed approach. It is noted that visitor origin is part of both the temporal and spatiotemporal analyses.

**Table 1:** List of analysis supported using our approach based on the parameter, description, and analysis dimensions.

Parameter	Description	Dimension
Day of the year	Provides the basis for monitoring visitor enjoyment (positive, negative, neutral) throughout the year. This parameter can capture special events and activities.	Temporal
Meteorological season	Daily average sentiment score is inspected based on its distribution across meteorological seasons.	Temporal
Day of the week	Daily average sentiment score is inspected based on its distribution across days of the week.	Temporal
Location Sentiment Progression	Sentiment over time based on an individual’s location.	Spatiotemporal
Enjoyment Measure	Sentiment score of a location category based on sentiment progression.	Spatiotemporal
Multi-Location Sentiment Progression	Sentiment over time based on multiple locations.	Spatiotemporal

### Dataset

**Fig 3:**
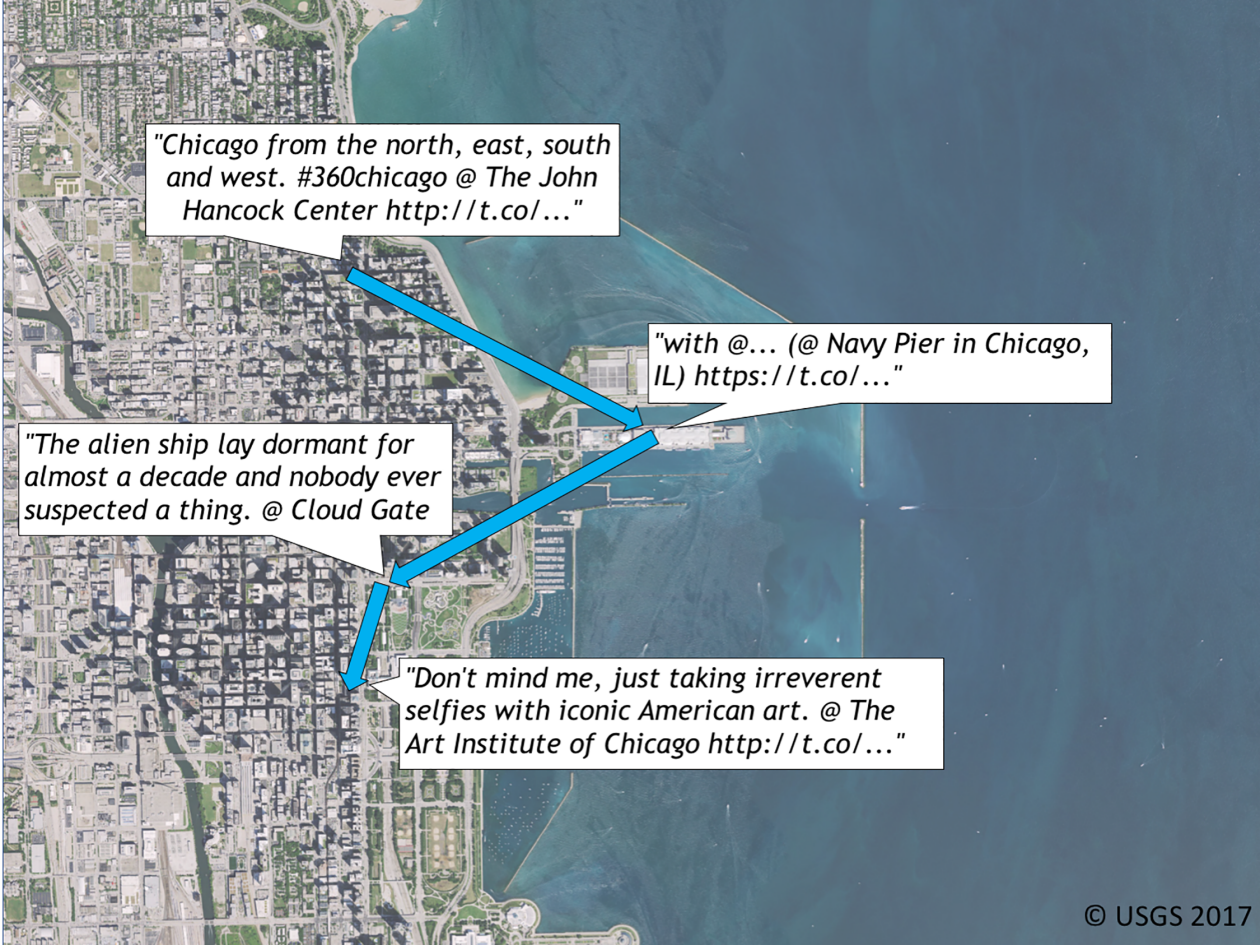
An example visitor trip. An example visitor from our Chicago attraction visit dataset visiting four places in a single day. We exclude names and personal identifiers.

We select Chicago, Illinois, USA as our use case city because it is a popular tourist destination. Tourist attractions in Chicago are closely located together with some even containing overlapping boundaries. As such, it provides a challenging test for our approach compared to cities with attractions that are spread apart. Overall, we identify 43 tourist attractions in Chicago (see S1 Table for our initial attraction list) and we collect 8,034,025 tweets from 225,805 users within Chicago’s boundaries and identify those tweets pertaining to attraction visits. Fig 3 illustrates an example visit sequence and its associated tweet texts of a tourist within our final dataset. S1 File shows the initial attraction visit dataset creation and cleaning procedures.

We assign people living in the state of Illinois or within the Chicago Metropolitan Area (which includes parts of the states of Indiana and Wisconsin) a *local* visitor origin type. S1 File provides detailed descriptions of our queries, common visitor location report methods, and the number of visitor origins identified. Final attraction visit numbers are provided in S2 Table as supplemental information. Table 2 shows the number of unique visitors and the number of total visits per visitor origin. *Locals* account for approximately 28% of visitors; 35% are *out-of-state*; 10% are *international*; and we classify 27% with *undetermined* origins. To better differentiate between visitors, we eliminate visitors with *undetermined* origins. This reduces the total visitors to 23,306 and total visits to 61,104. The final visit data is anonymized and made available in S1 Dataset.

**Table 2:** Number of visits and visitors by visitor origin.

	Number of visitors	Visitor percentage	Number of visits
*Local*	8,907	27.9	29,084
*Out of state*	11,246	35.2	23,611
*International*	3,153	9.9	8,409
*Undetermined*	8,618	27.0	18,080
**Total**	**31,924**	**100**	**79,184**

We show the number of visitors and the number of visits per attraction category in Table 3. Visits vary by attraction category where visits to places like gardens and observatories are less common than places like parks and museums. We note that the number of visitors for attraction categories (36,289) exceeds the overall number of visitors (23,306). This discrepancy is present because the visit numbers in each attraction category are calculated separately and a single visitor can visit multiple attractions.

**Table 3:** Number of attraction visits and visitors by attraction category.

Attraction category	Number of visitors	Number of visits
Aquarium	1,336	2,047
Architectural Beauty	950	1,620
Beach-Walking Area	3,074	4,916
Garden	466	854
Landmark	5,278	9,494
Museum	5,384	10,844
Observatory	634	1,170
Park	5,690	10,103
Shopping	1,974	2,975
Statue	4,974	6,463
Tower	4,625	7,342
Zoo	1,904	3,276
**Total**	**36,289**	**61,104**

## Results

### Temporal analysis

**Fig 4:**
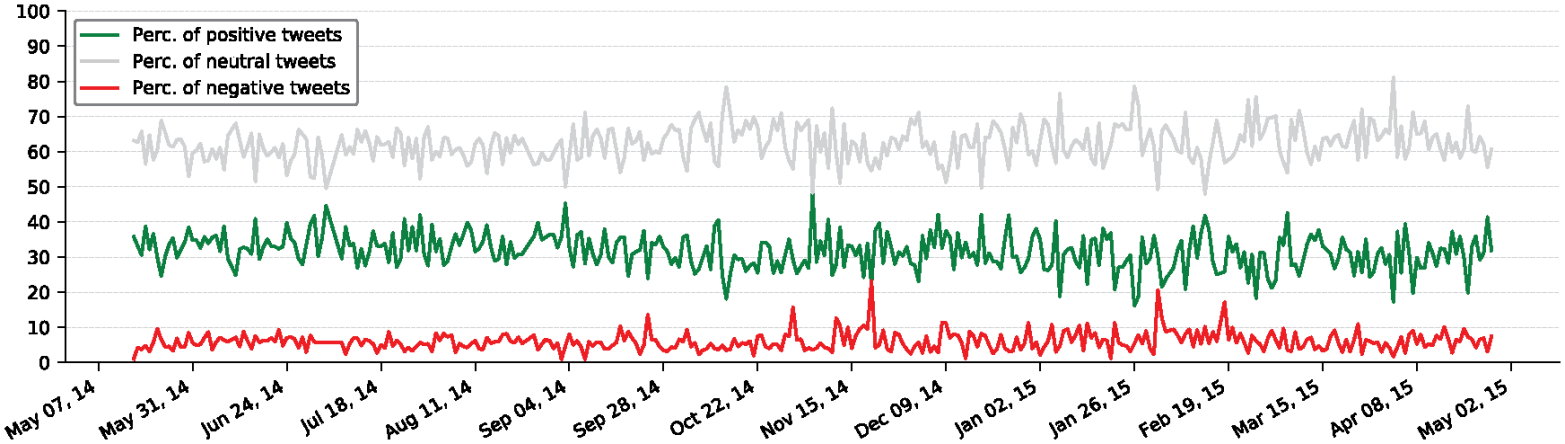
Day of the year sentiment patterns. Daily negative, positive, and neutral sentiment percentages spanning across a year. The data is generated for days with at least 50 attraction visits. The sentiment percentage trends here are quite consistent with some outliers that provide insight into why such outliers occur.

Temporal analysis (pattern identification of time-based sentiment) provides information of what impact, if any, **day of the year**, **season**, or **day of the week** has on visitors’ sentiments. Fig 4 shows the percentage of daily tweets separated into sentiment categories. The trends in the figure resemble a normally distributed sentiment percentage centered on the mean values (see S1 File). Assuming that the normal distribution seen in data is the expected quantity, we can focus on positive and negative outliers that can make a connection between day of the year and sentiment percentages. We identify three main patterns for sentiment pertaining to the day of the year.

The most salient pattern reflects city-wide *weather-related negative feelings*. Starting in late November, negative feelings about cold weather and snow are expressed on several occasions. These feelings become even more prevalent during the February blizzard in Chicago.

**Fig 5:**
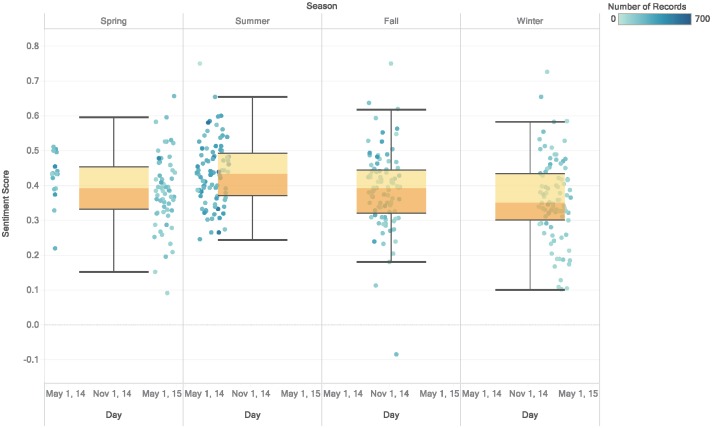
Sentiment scores per season. Daily average sentiment scores split across four seasons from Spring to Winter. The data distribution in each season is summarized using a Box and Whisker plot. Summer receives the highest positive sentiment with many data points rising above a 0.4 score. Spring and Fall follow with similar sentiment scores concentrated below 0.4 scores. Winter, on the other hand, appears to have the lowest sentiment scores with a concentration falling below 0.35.

Inspection based on seasonality reveals a number of additional insights. Fig 5 shows that summer ranks the highest among the distribution of average daily sentiment scores for all seasons. The spring and fall seasons have very similar distribution shapes coming after the summer season. The winter sentiment distribution skews lower than the other three seasons. The seasonal distribution of sentiment scores following seasonal temperatures confirms the findings of [42] who shows a direct correlation between sentiment scores and weather. Further, the uniformity of the score distributions within each season indicates the consistency of its effect within the season.

Other identifiable patterns are *special day* and *seasonal events*. Days such as the Fourth of July and Halloween and seasonal events such as summer concerts, Christmas lights, and ice skating receive positive opinions. We note that while Halloween is a fun event, its associated terms, like horror, provide a sense of negative sentiment. We also notice the existence of days associated with highly positive feelings that lack a significantly identifiable event or item. We provide three word-clouds [45] that represent one example per theme in S1 File.

Considering temporality and visitor type, we find that seasonal trends hold for different visitor types and different time periods within the day. We find very similar seasonal trends across different visitor types but we notice that the magnitude of scores are quite different. *Locals* and *out of state* visitors have very close seasonal numbers revolving around 0.38 and 0.45 median scores. *Internationals*, however, have approximately 25-30% lower scores than the others. These findings indicate that the seasonality in sentiment are present in different visitor types while the intensities vary between domestic (local and out of state) and international visitors. When we initially compare visitor types only, we notice that the difference in the magnitude is due to the high presence of neutral sentiment (i.e., lower average score) among international visitors. These points are showcased in S1 File. When looking at the relationship between seasonality and periods of the day (i.e., morning, afternoon, and evening/night), we discern no consistent pattern.

**Fig 6:**
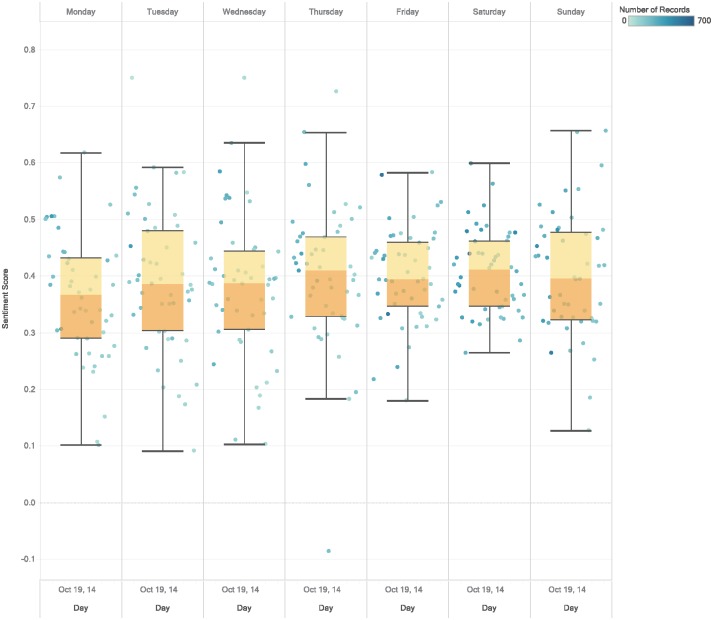
Sentiment scores based on the day of the week. Daily average sentiment scores split across the days of the week from Monday to Sunday. The data distribution in each day is summarized using Box and Whisker plots. The median score for all days ranges between 0.36 and 0.41. The upper and lower Whisker distances are quite large (0.33 to 0.64) indicating the variability of the scores in different weeks.

Fig 6 shows that sentiment scores are close in variations and percentiles when analyzed by day of the week. Thursdays and Saturdays have slightly higher sentiment scores and Mondays have slightly lower sentiment scores. To better understand the similarities and differences of the day of the week sentiments, we investigate these sentiment scores with respect to other factors including day period (morning, afternoon, night), season, and visitor type. We discern no consistent relationship between the day of the week and any of these factors.

**Fig 7:**
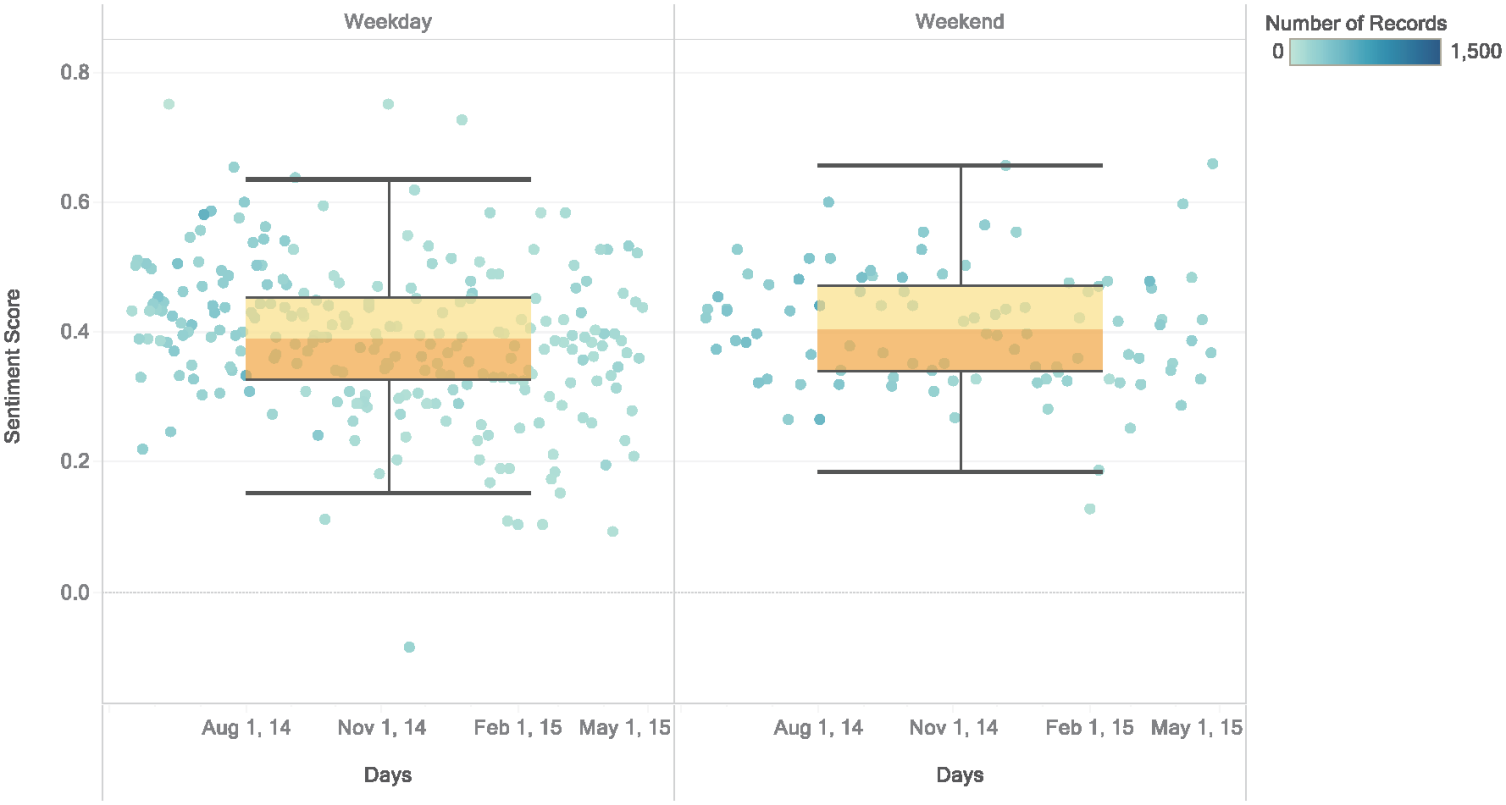
Sentiment scores based on weekday/weekend. Daily sentiment scores grouped by weekdays and weekends. The scores appear to distribute evenly, but with slightly higher scores for weekends.

By grouping days into weekdays and weekends (Fig 7) we find no real difference between these two groups. However, when considering seasons, **weekends** have relatively higher scores in all seasons except for summer. Summer has a better sentiment score for weekdays (see S1 File). These findings suggest that attractions are enjoyed more during summer **weekdays** while the opposite holds for all other seasons. The reason could be related to the fact that people take vacations in summer and good weather positively influences peoples’ moods. When looking at visitor types, we find no difference between weekdays and weekends. The same consistency appears in the sentiment scores for the period of the day versus weekday/weekend. This means that weekend/weekday sentiments only vary seasonally.

### Spatiotemporal analysis

We explore whether visitors’ feelings change or remain consistent over the duration of a visit. This identification is important for assessing sentiment per category over time. We identify this by using a subset of the attraction visit dataset that includes visitors with at least two consecutively shared tweets on the same-day originating from the same attraction. A total of 24,230 tweets from 8,401 visits made by 5,818 visitors satisfy this criterion. We limit our analysis to the tweets shared within the first four hours corresponding to 22,817 tweets. We investigate the progression of sentiment over time for visits starting with positive, neutral, or negative sentiment separately to observe sentiment progression.

**Fig 8:**
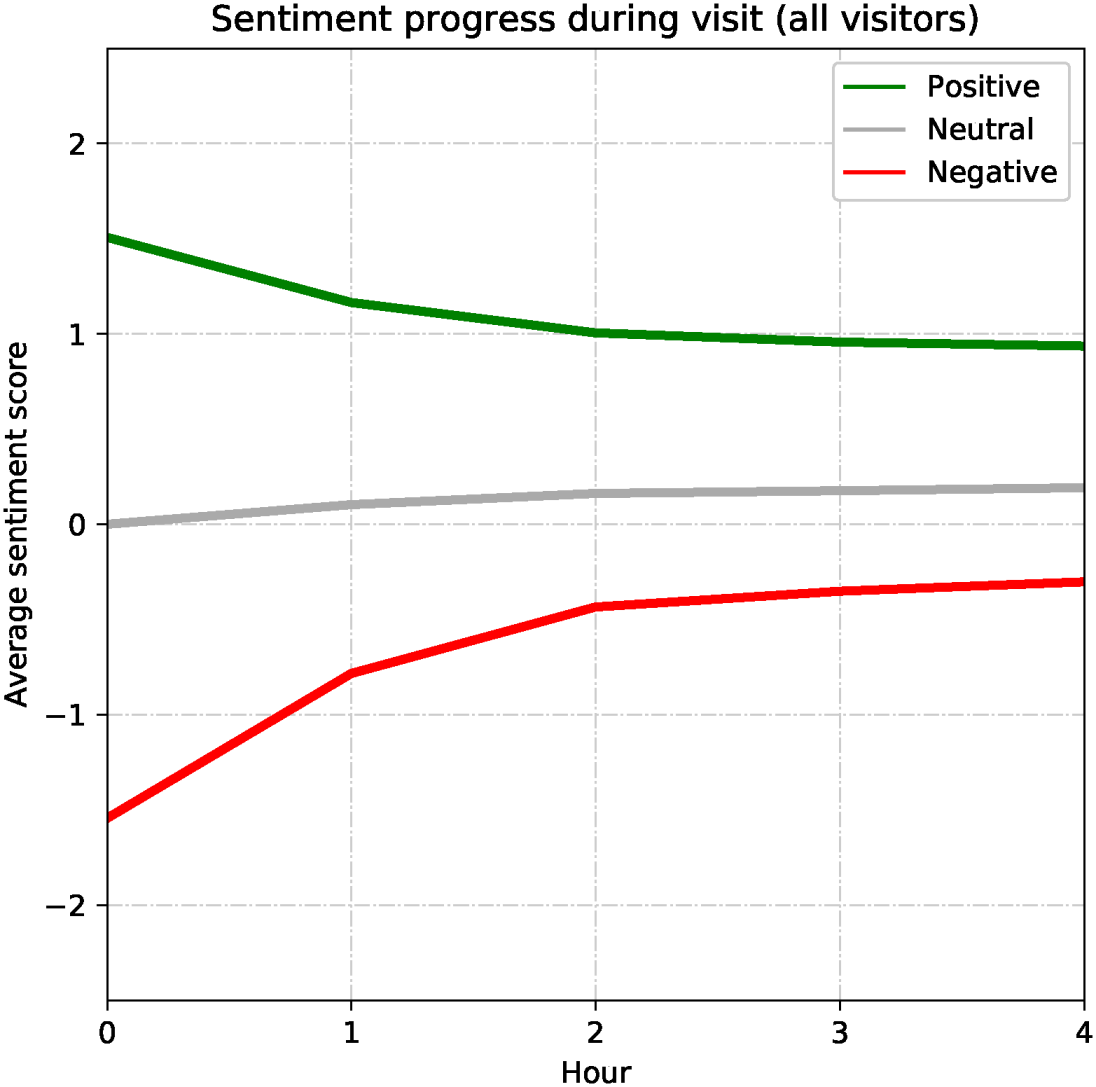
Sentiment progression based on visit length. A plot of the progress of sentiment based the time spent at an attraction. This graph shows all visits combined to reflect general behavior seen in attraction visits. Green line (upper) shows positive, gray line (middle) shows neutral, and red line (lower) shows negative sentiment progression.

Fig 8 illustrates positive, neutral, and negative **location sentiment progression** patterns based on all visits combined. These patterns describe a general perspective on visitor sentiment progression. In the graph, the initial sentiment strength of both positively and negatively started visits are around the scores of 1.5 and -1.5, respectively. Then, both scores approach neutral sentiment at different rates. The initially positive visits become less positive within the first two hours but then remain consistent around the score of 1 until the end of the four-hour period. On the other hand, the initially negative visits transition more quickly towards neutral and remain consistent around the score of -0.3. The initially neutral visits become slightly positive by their end. These numbers describe general visit enjoyment trends with respect to visit length. We observe almost identical patterns for different visitor types.

**Fig 9:**
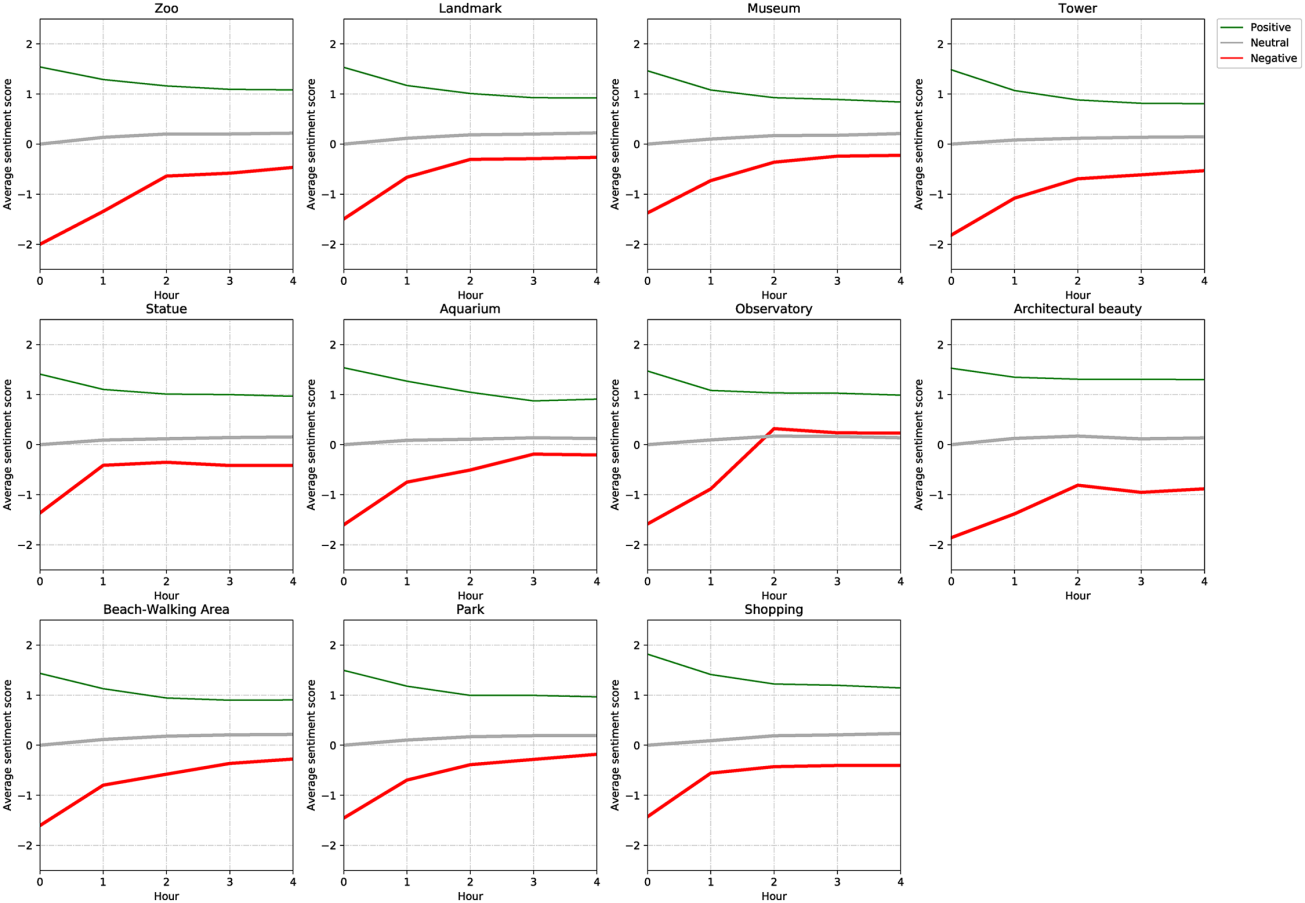
Sentiment progression based on visit length and attraction category. A plot of the progress of sentiment based the time spent at an attraction. These graph shows visits grouped by attraction category. Color coding and placement is the same as the previous figure.

Looking for patterns of different attraction types, a visual inspection on Fig 9 shows the positively-started visits have similar trends to the overall case (Fig 8) with sentiment starting around 1.5 and ending around 1.0 after a four-hour period. Categories of shopping and architectural beauty end around 1.2 while the tower category sentiment ends around 0.8. The end scores may indicate the level of interest for these attractions among people who already like them. The neutrally-started visits show almost identical trends by slightly climbing to the positive sentiment side. This may suggest that visitors with no strong initial feelings about any attractions end up with some positive feelings. The negatively-started visits, on the other hand, vary the most. Visits to the zoo, architectural beauty, and tower categories start very negatively about -1.9 while the other category visits start around -1.4 to -1.5. A majority of initially negative category visits end with a sentiment score around -0.5. This suggests that visitors who initially dislike places end up feeling less negative. Observatory visits, unlike others, jump into the positive side in the end suggesting that people end up liking observatory places regardless of initial feeling. Negatively-started architectural beauty visits end quite negatively around -1.0 indicating that four hours is not long enough for people to gain a new appreciation of this attraction category.

**Fig 10:**
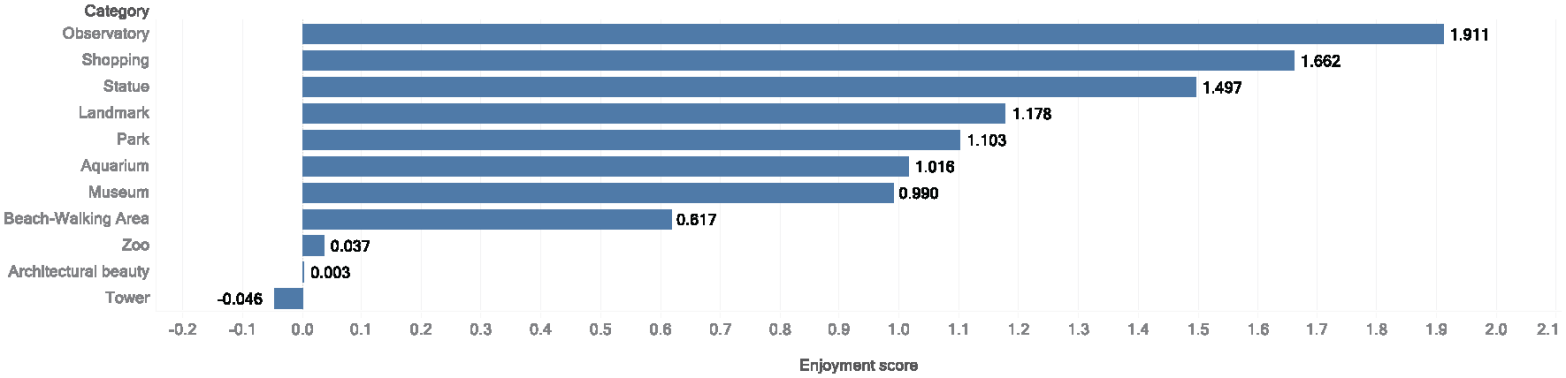
Enjoyment measure scores. Enjoyment score of attraction categories calculated according to the *f*_*em*_ function.

We apply the enjoyment measure function, *f*_*em*_, to calculate the enjoyment score for each attraction category. Fig 10 shows the Observatory category has the highest enjoyment score as it is the only category where positive and negatively started visits end with positive sentiments. The Shopping category comes after that because of the persistent high positive score (1.0>) and relatively lower magnitude of negative scores. The last three spots are taken by the Zoo, Architectural Beauty, and Tower categories, respectively. We note that the neutral sentiment trend is not counted in this function because it does not vary much by attraction category.

**Fig 11:**
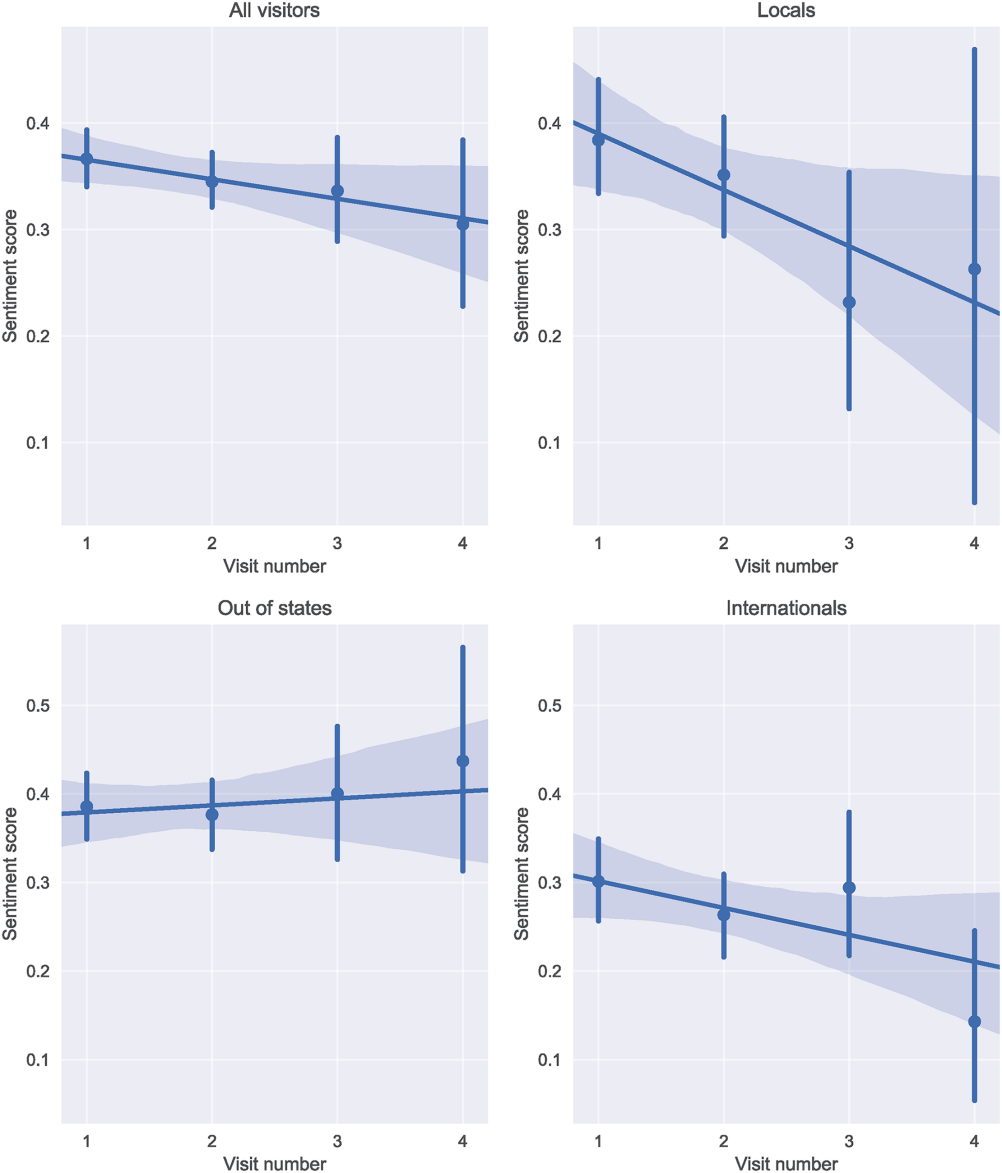
Multiple visit scores for overall visits and based on user type. Linear regression models are fit according to the average sentiment score at attraction visits vs. visit number in the day. The first graph in the first row is gathered from the entire user set whereas the following three graphs are based on different visitor origins. Shaded areas around the linear regression line contains the 95% confidence interval and the round markers in each visit number marks the average sentiment score for that visit. We note that all regression fits are statistically significant.

The last pattern that we investigate is sentiment when visiting different attractions in a day (**multi-location sentiment progression**). We hypothesize that sentiment changes (positively or negatively) after several places are visited in a single day. As such, we look at visitors who visit more than one attraction in a day. We identify 13,907 attraction visits from 3,027 users with at least two attraction visits on the same day. To see the statistical differences between these visits, we plot the linear regression fit in Fig 11 based on the average visit scores in multiple visits against the visit number. In general, there is a slight decreasing sentiment trend when people visit multiple locations. When we break it down by visitor origin, we find a sharper decrease for locals. This could be related to the fact that locals are already familiar with these places and more visits make their enjoyment less interesting. The international visitors’ trend is also on the decreasing side. While this trend is not as dramatic, it appears that the first three visits are enjoyed quite equally whereas the fourth one is definitely on the lower side. This could be related to the tiredness of long trips that internationals take. Surprisingly, multiple visits do not negatively influence out of state visitors. These attractions are still novel for out of state visitors and, generally, out of state visitors do not have the long trips experienced by international visitors to reach the attractions.

With the multi-location sentiment progression inspection, we conclude our analysis. We summarize our results collectively in Table 4. Using the Chicago use-case dataset, we are able to gather insights into all of the parameters we investigated.

**Table 4:** Summary of insights obtained from the Chicago dataset based on the parameters identified in our approach.

Parameter	Insight from Data
Day of the Year	Weather-related negative sentiment is noticeable. Special days like the 4th of July and Christmas and events like concerts and soccer game displays generate positive tweets.
Meteorological Season	Summer visits are enjoyed the most while winter visits are enjoyed the least.
Day of the Week	Weekdays are enjoyed more during the summer while the opposite holds for other seasons.
Location Sentiment Progression and Enjoyment Measure	Visitors experience greater enjoyment in places like observatories than zoos.
Multi-Location Sentiment Progression	Generally, multiple visits in a day result in decreasing sentiment holding for local (steeper decrease) and international visitors. Out of state visitors tend to have better sentiment when visiting multiple attractions on the same day.

## Conclusion and discussion

In this paper, we presented an approach using Twitter data to investigate the relationship between tourists’ feelings on their attraction visits in a city by investigating the temporal and spatiotemporal dimensions of their tweets. The approach comprises four major steps. First, data collection and preprocessing create an attraction dataset and capture and clean a visit dataset from Twitter for a selected city. Second, visitor identification takes place that labels users’ origin categories based on their self-reported locations. Third, positive and negative emotions in tweets pertaining to attraction visits are assessed and assigned numerical scores. Fourth, attraction visit tweet sentiments are investigated based on their temporal and spatiotemporal dimensions and insights from these investigations are reported. The temporal and spatiotemporal dimensions include: day of the year; meteorological season; day of the week; location sentiment progression; enjoyment measure; and multi-location sentiment progression.

Applying the approach to the City of Chicago, we identify that season plays a major impact on tourist sentiment. The city of Chicago is known for harsh winters and negative tweet sentiment appears as a result. This assessment can extrapolate to other cities where climate patterns, such as heavy rain, play a role. This should assist planners and decision makers in determining what tourist activities should be developed or promoted within those climates. Special days like the 4th of July and Christmas and events like concerts receive positive feelings from visitors. Weekdays are enjoyed more during the summer while the opposite holds for other seasons. Analysis suggests that attractions like observatories and statues receive greater sentiment than zoos or architectural beauty. Finally, we find that local and international visitor tend to have negative sentiment when they visit more than one attraction in a day whereas the opposite holds for out of state visitors.

There are several advantages of our study for researchers and practitioners. Our proposed approach provides the means for investigating tourist emotions at a very granular level that captures visit time, visitor origin, attraction name, and attraction category for a long period of time. To our knowledge, no other approach provides tourist emotions at this granularity. Furthermore, our approach is designed to be generalizable for use with any city around the world. Finally, we use Twitter data in our analysis which is freely available for the public.

There are some limitations that can be addressed in future studies. For instance, our first step involves the creation of an attraction list and the collection of an attraction visit dataset which requires manually checking online sources and identifying attraction boundaries and keywords. The attraction list can potentially be obtained automatically from online sources like Google Places and Foursquare and the corresponding attraction boundaries can be created using building footprints from official sources or creating a circular boundary based on the center point of the attraction along with its type. The determination of visitor origin in our approach makes use of the location property of users. We could also use other Twitter messages of the person and infer hometown based on the concentration of tweet content words [22]. The sentiment analysis method that we adopt does not account for sarcastic statements that sometimes exist in social media posts; however, this is a challenging task even for manual human identification.

Like most studies that rely on social media data, major limitations come from how representative social media data is of a population. In the presented use case, the streaming API of Twitter allows collecting only up to one percent of the entire tweet stream. While there is no systematic bias on the selection of one percent of tweets, there is a non-transparent mechanism on the streaming API that provides statistically similar samples to different consumers when they try to consume the API with the same filtering parameters [46]. This selection mechanism applies to our data collection as we also use the Streaming API. In addition to the selection challenge, the representativeness of the Twitter population is a limitation even when representing stable populations with well-known characteristics [47]. Capturing the characteristics of tourist populations is usually made at high-level using travel number from other regions and countries. Visit numbers frequently change based on the economy, safety, and infrastructure in origins and destinations. This leaves us with a limited comparison point between the Twitter population and actual tourist population. We compare our Chicago dataset visitor origin percentages (≈86.5% US visitors vs. ≈13.5% internationals) with actual visitor percentages for the data collection years (≈97% US visitors vs. ≈3 internationals) [48]. Our visitor base over-represents the international population. We note that our dataset includes only visitors to popular Chicago attractions which is both a subset of all visitors as well as a subset of all attractions. These attractions, in particular, may attract international visitors at a relatively higher frequency than US visitors. Thus, the bias may be smaller than indicated. Future studies will address the aforementioned challenges.

## Supporting information

S1 Dataset Visit dataset. This dataset contains anonymized visit information including visit time, visited attraction name and category, visitor anonymized ID, visitor category, and sentiment score.
(CSV)

S1 Table Initial tourist attraction list for the city of Chicago. The list contains attraction’s name, category, rankings in online tourist platforms, boundary-based and distance-based keywords, opening and closing hours, scores from online platforms, and boundary points. We only consider one opening and one closing hour per attraction that covers the earliest opening hour and the latest closing hour considering all business hours within the year. Also, opening hours are rounded down and closing hours are rounded up to the closest integer.
(CSV)

S2 Table Final tourist attraction list with number of visits. The final list of tourist attractions and corresponding visit numbers identified within our datasets. We eliminated visits made by undetermined origins that either have empty or unidentified location information. Eliminating these tweets provided a better differentiation between visitor types in the final dataset.
(CSV)

S1 File Supplemental information. (PDF)
