# Supplementary material for: Temporal and spatiotemporal investigation of tourist attraction visit sentiment on Twitter
Source: PLoS One. 2018 Jun 14;13(6):e0198857. doi: 10.1371/journal.pone.0198857 (PMC6002102; doi:10.1371/journal.pone.0198857)
Supplement: S1 File — (PDF) [file pone.0198857.s004.pdf]

Temporal and Spatiotemporal Investigation of Tourist Attraction Visit Sentiment on Twitter

Supplemental Information

Jose J. Padilla[1], Hamdi Kavak[1,2], Christopher J. Lynch[1], Ross J. Gore[1], Saikou Y. Diallo[1]

hkava001@odu.edu

[1] Virginia Modeling Analysis and Simulation Center - Old Dominion University, Suffolk, VA

[2] Modeling Simulation and Visualization Engineering Department - Old Dominion University, Norfolk, VA

1 Identification of initial attraction visit dataset

Our entire Chicago visit dataset contains 8,034,025 geo-located tweets originating from 225,805 users collected between May 16, 2014 and April 27, 2015 (missing four days). First, we applied boundary-based identification by finding tweets located within an attraction's boundaries that contain at least one keyword related to that attraction within their texts. In this way, we identified 67,737 attraction visit tweets from 30,574 visitors. Second, we gathered tweets that are shared in six hours of an attraction visit while the visitor is still within the attraction's boundary. In this way, we identified an additional 8,630 tweets from 3530 visitors. Finally, we applied distance-level identification by selecting tweets containing the attraction's full name within the tweet text and located within a one kilometer distance of the attraction's boundary. With this step, we gathered another 5,579 attraction visit tweets from 4248 people. Visit data is shown in Table 1. We eliminated two attractions due to low numbers of tweets (< 50). In total we gathered 81,908 attraction visit tweets from 32,559 unique visitors.

2 Cleaning the outliers in the initial attraction visit dataset

In order to make sure that we only gathered visit related tweets, we aimed to eliminate outliers from the initial visit tweets based on time of tweeting. Fig 1 illustrates how Chicago attraction visits are distributed over the course of the day. According to the figure, a majority of attraction visits occur between 9AM and 11 PM while the peak attraction visit timeframe occurs between 1PM and 4PM. This result intuitively reflects real-world attraction visit patterns where many attractions are open within these times. Attraction visits tweets also follow a quite different pattern than both general Chicago or USA tweets, in that many of the tweets are shared after 5PM until midnight. This is a positive indication that tweets from attraction visits are distinct from general tweets.

To ensure that the temporality of attraction visits aligns with attractions' operating hours, we use the opening and closing hours of each attraction gathered while compiling the attraction dataset. Excluding attractions that are open 24 hours a day, we filtered the tweets shared outside of business hours of each attraction. We assume visitors can arrive an hour earlier than the opening hour and can

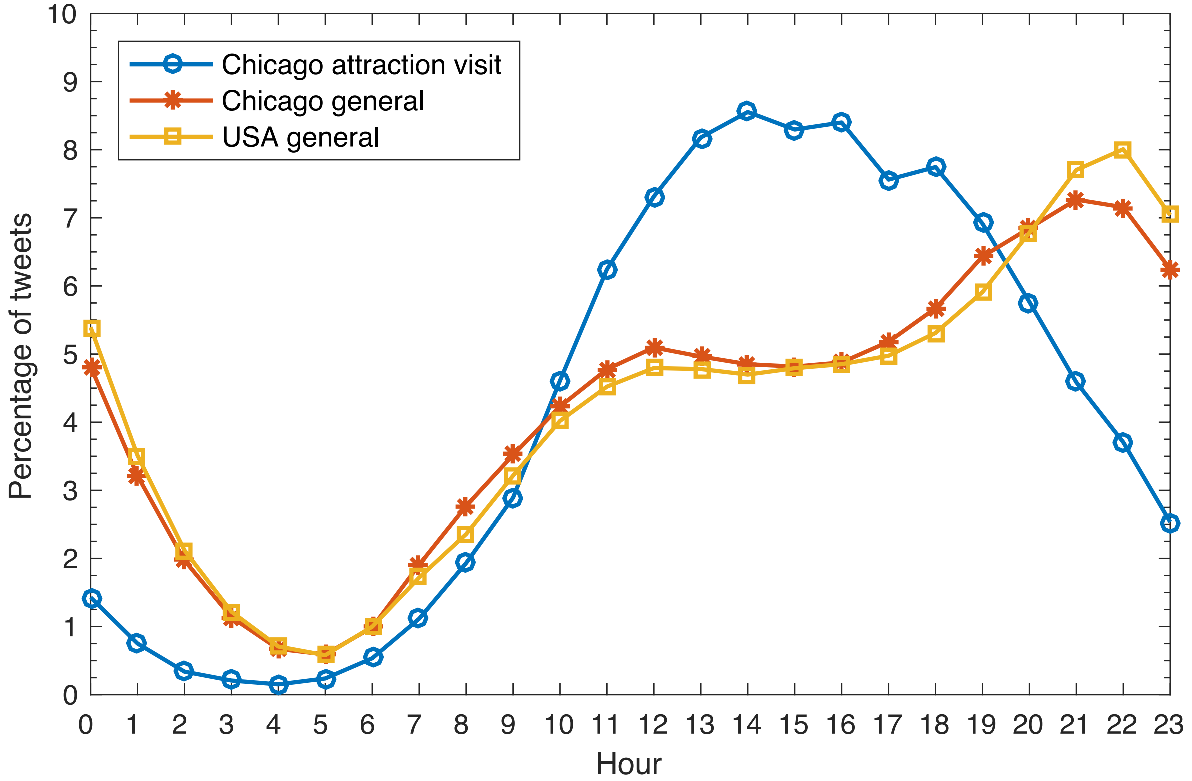

Figure 1: Hourly tweet distribution of attractions visit in Chicago against general hour distributions gathered from general Chicago geo-located dataset and general USA geo-located dataset.

Table 1: Tourist attraction list with number of visits arranged alphabetically. Attractions highlighted in gray are eliminated due to low numbers of tweets.

**Attraction**	**Tweets**	**Attraction**	**Tweets**
360 Chicago Observation Deck - John Hancock Center	3,155	Museum of Contemporary Art Chicago	1,688
Adler Planetarium	1502	Museum of Science and Industry	1731
Buckingham Fountain	1461	National Museum of Mexican Art	316
Chicago Children's Museum	198	Navy Pier	9778
Chicago Cultural Center	1248	North Avenue Beach	2972
Chicago History Museum	383	Oak Street Beach	1338
Chicago Riverwalk	2118	Oriental Institute Museum	52
Chicago Sports museum	141	Picasso Statue	146
Cloud Gate	8672	Richard H. Driehaus Museum	74
Crown Fountain	319	Robie House	64
Flamingo Sculpture	64	Rockefeller Memorial Chapel	101
Garfield Park Conservatory	526	Rookery Building	91
Graceland Cemetery	93	Shedd Aquarium	2798
Grant Park	3887	Skydeck Chicago - Willis Tower	7079
Historic water tower	188	The Art Institute of Chicago	6027
Holy Name Cathedral	169	The Field Museum	3147
Lincoln Park Conservatory	417	The Magnificent Mile on Michigan Ave	2836
Lincoln Park Zoo	4070	The McCormick Bridgehouse & Chicago River Museum	1
Lurie Garden	175	The Peggy Notebaert Nature Museum	231
Maggie Daley Park	690	Tribune Tower	919
Michigan Avenue Bridge	587	Water tower	1701
Millennium Park	8418	Wrigley Building	338
Money Museum at the Federal Reserve Bank	37		

leave one hour later than the closing hour. We found 2,724 outliers (≈3.3%) that are shared outside of attractions' open hours. In the end, we obtained 79,184 total attraction visits from 31,924 visitors.

3 Identification of visitor origin

As mentioned in the main text, we used location information provided in Twitter profiles of visitors contained in the attraction visit list. Table 2 shows the top 30 most commonly reported location information terms used within their profiles. Almost 21% of visitors provided no location followed by a relatively large Chicago/Illinois-related location information. The remainder of the location information mostly refers to major US cities and states. In total, we found 10,615 case-insensitive unique location information from 31,924 visitors.

We apply the two-step visitor origin identification approach to match location information with one of the three visitor origin categories. In the first step, we identify local and out of state visitors providing structured location information. We constructed queries provided in Table 3 to mark these visitors. For the remaining 8,721 visitors, we used Google Maps API to identify their corresponding visitor origins.

Table 2: Top 30 commonly used case-insensitive location info by the visitors. Number of appearance is given in the second column.

**Location**	**Count**	**Location**	**Count**	**Location**	**Count**
	6816	michigan	97	nashville, tn	74
chicago	2836	usa	95	chicago,il	74
chicago, il	2620	chicago il	95	boston, ma	71
chicago, illinois	316	washington, dc	92	milwaukee, wi	69
new york, ny	195	san francisco, ca	90	brooklyn, ny	64
los angeles, ca	153	illinois	88	austin, tx	63
los angeles	151	toronto	87	madison, wi	61
new york	132	minneapolis, mn	81	indianapolis, in	56
new york city	129	san francisco	81	m?xico	56
nyc	117	atlanta, ga	77	london	54

Table 3: Visitor origin identification queries.

**Query**	**Mark as**	**Matching visitors**
Contains *'chicago'* or *'chi'* as a word	Local	7,659
Contains *'one of the county names in Chicago Metropolitan Area'* as a word and does not contain *'state names or abbreviations accept for Illinois, Indiana, and Wisconsin'* as a word	Local	268
Contains *'il'*, *'il'*, or, *'illinois'* as a word	Out of state	499
Contains the patterns of *', state name/abbreviation'* or *'state name/abbreviation'* (except for Illinois) and does not contain certain country names that conflicts with state name/abbreviation patterns.	Out of state	6146
Contains certain major us city names except for Chicago	Out of state	1815

4 Other patterns

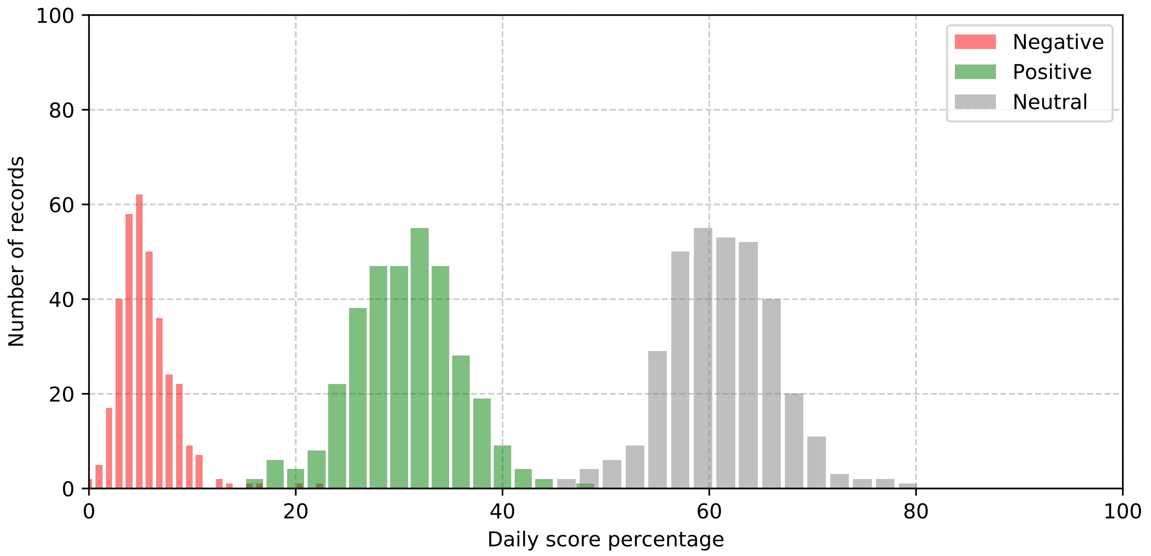

Figure 2: The distributions of daily sentiment percentages for three sentiment polarity values. All the distributions resemble Gaussian-like shapes. The positive and negative sentiment percentages span on a wider curve whereas the negative sentiment percentage curve is narrower.

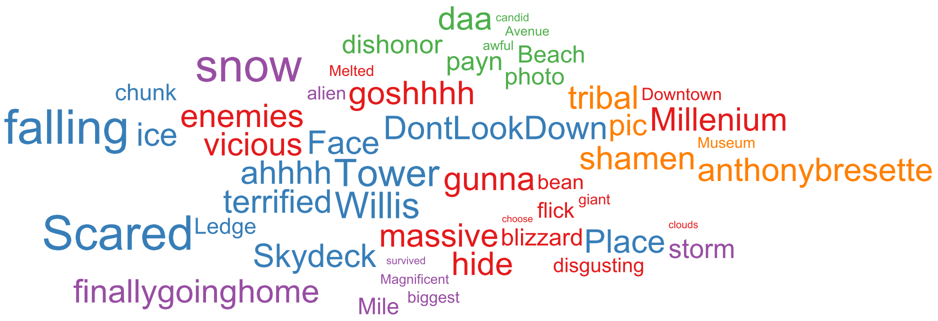

(a)

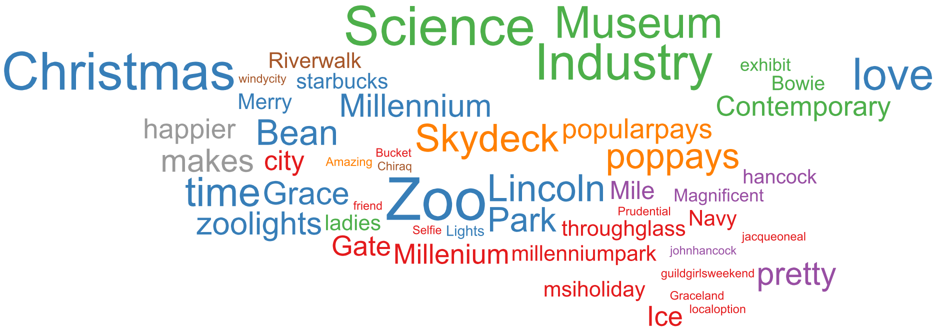

(b)

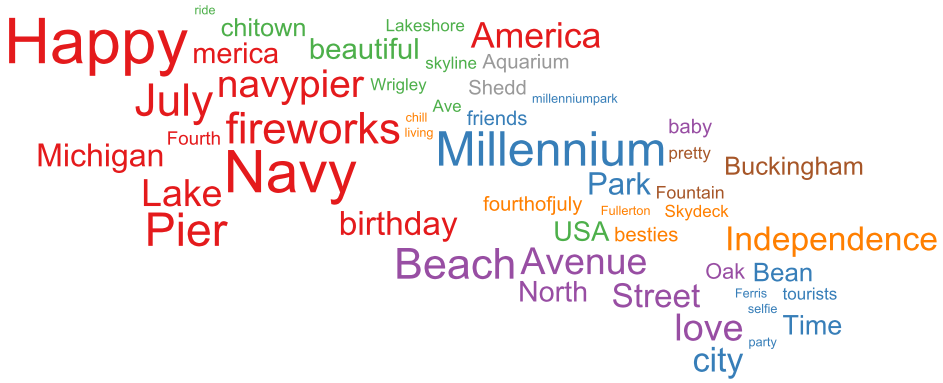

(c)

Figure 3: Word clouds generated for the day of the year patterns. **(a)** weather-related negative tweets with words like massive blizzard, falling ice, and snow - February 02, 2015. **(b)** Christmas, Zoo, and Lights related positive tweets seen during the Christmas season - December 07, 2014. **(c)** The US Independence Day celebration related positive tweets from the Navy Pier, Lake Michigan, and Millennium Park- July 04, 2014. We modified the word cloud generation algorithm to account for Twitter jargon (e.g., hashtags) and increase the dictionary for stop-words. We set word clouds output to a maximum of 50 words to maintain readability.

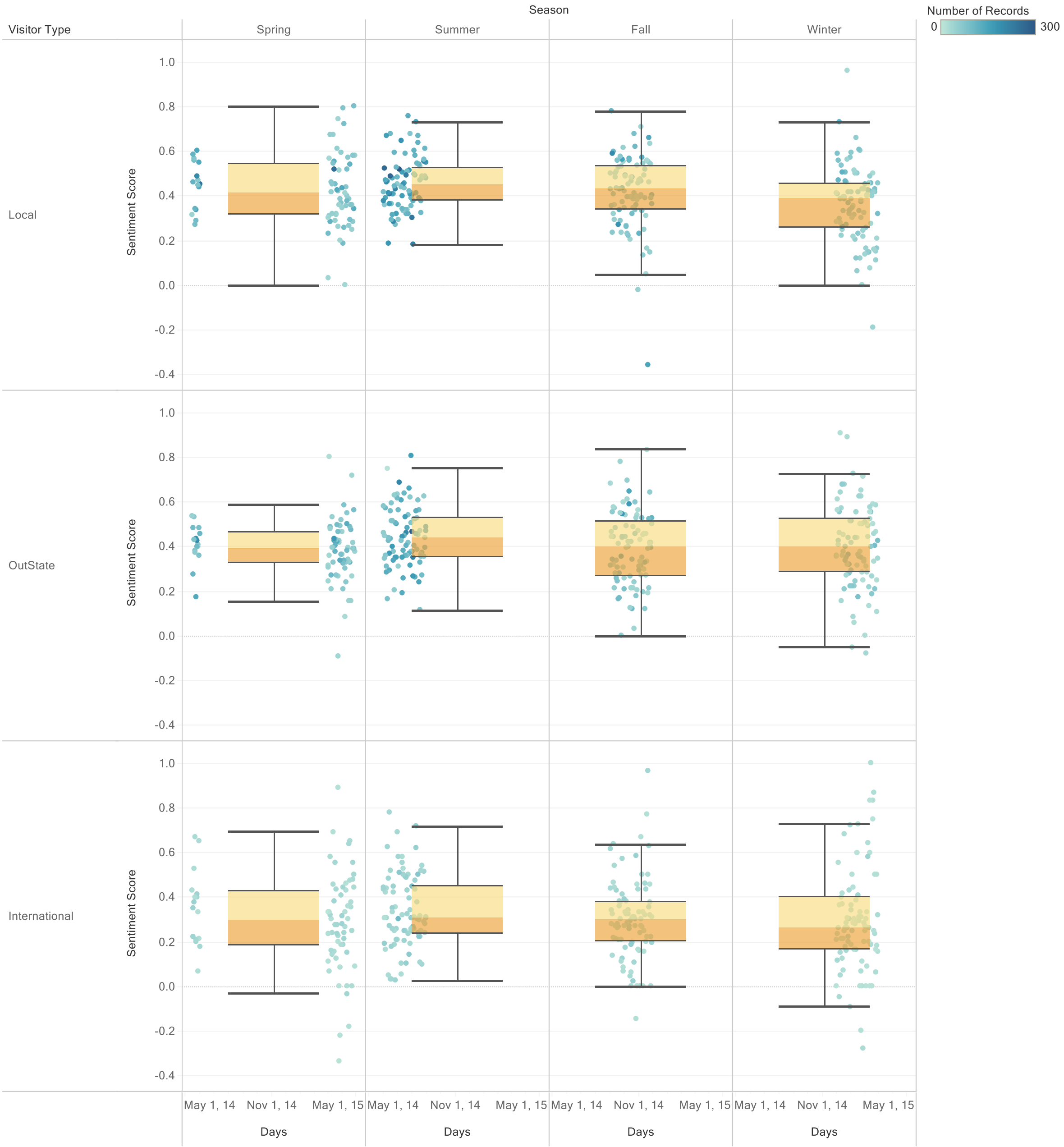

Figure 4: The distribution of average daily sentiment values split into four seasons and three visitor types. All visitor types seem to follow the same seasonal sentiment trends explained in the main text. The primary difference is on the magnitude of these scores where internationals have lower median scores than the other two.

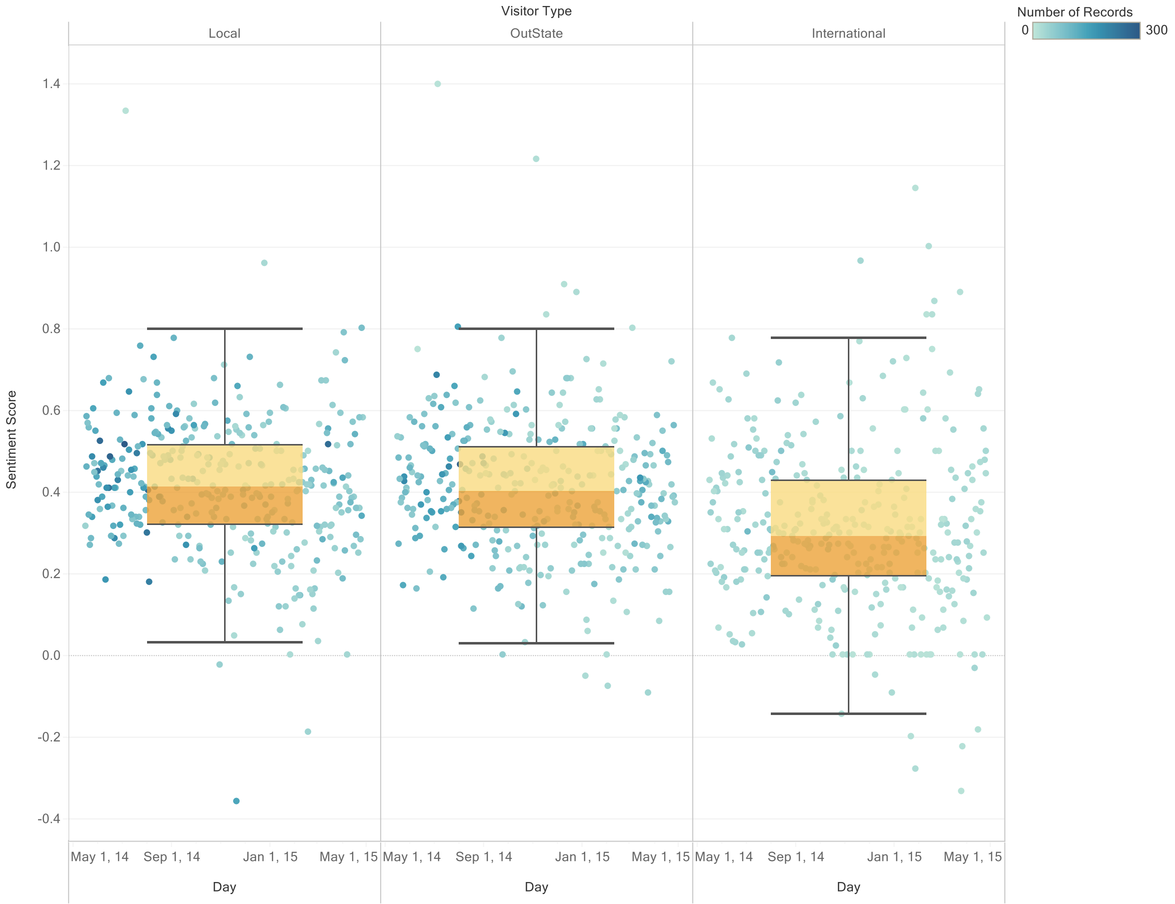

Figure 5: The distribution of the average daily sentiment values across three visitor types. Local visitors and out of state visitors have very similar score distributions that are relatively higher than international visitors. Looking at the high-level statistics, we noticed that international visitors tend to express neutral sentiment most of the time making their scores lower.

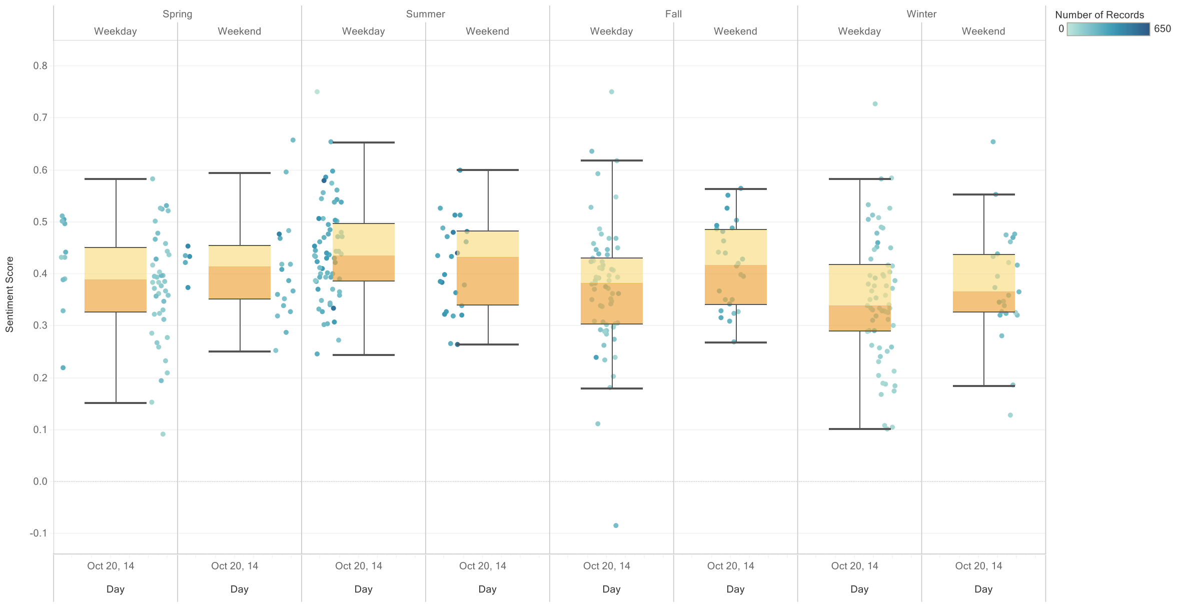

Figure 6: The distribution of average daily sentiment values based on season and weekday/weekend. Except the summer, weekends have greater enjoyment than weekdays. During the summer, weekdays have greater sentiment scores.
